# Comparison of psychometric properties between recall methods of interview-based physical activity questionnaires: a systematic review

**DOI:** 10.1186/s12874-019-0684-1

**Published:** 2019-03-01

**Authors:** Kenji Doma, Renée Speyer, Lauren Alese Parsons, Reinie Cordier

**Affiliations:** 1 College of Healthcare Sciences, James Cook University, Townsville, Queensland Australia; 2 Department Special needs Education, University of Oslo, Oslo, Norway; 3 School of Occupational Therapy, Social Work and Speech Pathology, Curtin University, Perth, Western Australia; 4 Department of Otorhinolaryngology and Head and Neck Surgery, Leiden University Medical Center, Leiden, the Netherlands

**Keywords:** Recall methods, Validity, Reliability, Direct measures, Indirect measures, COSMIN

## Abstract

**Background:**

This systematic review examined the methodological quality of studies and assessed the psychometric qualities of interview-administered Past-week and Usual-week Physical Activity Questionnaires (PAQs). Pubmed and Embase were used to retrieve data sources.

**Methods:**

The studies were selected using the following eligibility criteria: 1) psychometric properties of PAQs were assessed in adults; 2) the PAQs either consisted of recall periods of usual 7-days (Usual-week PAQs) within the past 12 months or during the past 7-days (Past-week PAQs); and 3) PAQs were interview-administered. The COSMIN taxonomy was utilised to critically appraise study quality and a previously established psychometric criteria employed to evaluate the overall psychometric qualities.

**Results:**

Following screening, 42 studies were examined to determine the psychometric properties of 20 PAQs, with the majority of studies demonstrating good to excellent ratings for methodological quality. For convergent validity (i.e., the relationship between PAQs and other measures), similar overall associations were found between Past-week PAQs and Usual-week PAQs. However, PAQs were more strongly associated with direct measures of physical activity (e.g., accelerometer) than indirect measures of physical activity (i.e., physical fitness), irrespective of recall methods. Very few psychometric properties were examined for each PAQ, with the majority exhibiting poor ratings in psychometric quality. Only a few interview-administered PAQs exhibited positive ratings for a single psychometric property, although the other properties were either rated as poor or questionable, demonstrating the limitations of current PAQs.

**Conclusion:**

Accordingly, further research is necessary to explore a greater number of psychometric properties, or to develop new PAQs by addressing the psychometric limitations identified in the current review.

## Background

The prevalence and severity of obesity is continually increasing in most of the Western world, developing into epidemic proportions worldwide [1]. Lack of physical activity reduces physical fitness, and is a major contributor to this global health crisis and is associated with development of chronic diseases and cancer, leading to increased mortality [2]. In contrast, participation in physical activity has been associated with improved health outcomes, lower incidences of health problems and reduced mortality rates [3–5]. International guidelines developed by the World Health Organisation (WHO) recommend that children and adults to engage in 60 min of moderate-to-vigorous physical activity each day [6, 7]. To assess whether physical activity is being performed at these recommended levels, adequate monitoring of patient’s lifestyles and behaviours is needed [8]. This enables health professionals to establish disease risks and develop interventions to address physical inactivity.

Questionnaires are typically used to assess physical activity level, as: 1) they are cost-effective and practical; 2) a large amount of information is collectable in a relatively short period of time; and 3) the results are easily quantifiable [9]. However, a number of disadvantages have been proposed, such as: 1) results are influenced by subjective measures; 2) misinterpretation of questions and recall bias due to language/cultural barriers or cognitive impairment; and 3) questionnaires not specifically developed for people with certain physiological/chronic conditions. Nonetheless, physical activity questionnaires are currently the most widely-used and acceptable forms of obtaining information on physical activity characteristics, particularly for larger-scale observational studies and research interventions [9]. There are several types of physical activity questionnaires which are primarily categorised according to recall periods. The two main recall methods currently utilised, measures recent physical activity performed over the past 7 days (i.e., Past-week PAQs) and the average week physical activity performed within the past 1–12 months (i.e., Usual-week PAQs) [10]. Previous research suggested that Past-week PAQs more accurately reflect the actual physical activity characteristics undertaken that week; however, Usual-week PAQs may minimise week-to-week variability [10], seasonal differences [11] and lifestyle factors, such as pregnancy [12]. Accordingly, the two recall methods may have distinct psychometric characteristics. It is therefore important to establish the validity and reliability of both types of PAQs, to ensure the PAQs selected are fit for purpose.

The Consensus-based Standards for the Selection of Health Measurement Instrument (COSMIN) checklist is a critical appraisal tool which evaluates methodological quality of studies that examine the psychometric properties of health related measures [13]. When combined with quality criteria for psychometric properties [14, 15], it provides a contemporary framework to assess overall psychometric quality of PAQs. According to a recent systematic review using the COSMIN checklist [16], when *convergent validity* was assessed by comparing PAQs with other measures (e.g., accelerometer or other PAQs), Past-week PAQs had higher correlations than Usual-week PAQs. These findings demonstrated that Past-week PAQs may assist clinicians in accessing the same constructs as those of other measures with better precision of PA level recordings. However, Doma and colleagues [16] only reported on studies that examined the psychometric properties of PAQs that were self-administered and excluded studies that administered PAQs via interviews. Whilst the ability to compare these data from self-administered PAQs to interview-administered PAQs are limited, it is currently the best available evidence of a similar construct.

The majority of PAQs can be either self-administered or interview-administered via face-to-face or telephone conducted by trained interviewers. For example, Active Australia Survey (AAS) is a commonly administered PAQ which assesses past-week PA level; its psychometric properties have previously been evaluated via both self-administration [17] and interview-administration [18]. The advantages of self-administered PAQs are that it is cost-effective, particularly when the PAQs distributed via postal mail or online, and minimises interviewer bias [19]. However, self-administered PAQs also risk introducing respondent bias, especially if respondents have literacy and numeracy difficulties [20]. These limitations can be overcome with interview-administered PAQs, although interviewees may overestimate reporting of their PA level due to social desirability [21]. In addition, the interviewee may over- or under-report physical activity level if instructions given by interviewers are not well standardised, or if interviewers are selective with phrasing the PAQs [22].

Although there is evidence that the mode of questionnaire administration may influence the accuracy and quality of the responses [20], to date, systematic reviews have only reported on the psychometric properties of self-administered PAQs [16, 23, 24], with overall findings indicating that only a few self-reported PAQs had reasonable reliability and validity ratings. No systematic reviews have explored the literature to determine the psychometric properties of interview-administered PAQs, particularly when compared between Past-week and Usual-week PAQs.

Therefore, the purpose of this systematic review was to evaluate the methodological quality of studies that have investigated the psychometric properties of interview-administered Past-week and Usual-week PAQs and to determine the overall psychometric quality for each PAQ. The results of this review will aid practitioners and researchers in selecting interview-administered PAQs that are appropriate for their purposes and through identifying the effects of recall differences on psychometric soundness.

## Methods

The current systematic review was conducted in accordance with the Preferred Reporting Items for Systematic Reviews and Meta-Analyses (PRISMA) statement [25]. The PRISMA statement is a checklist that consists of 27 items that are used to ensure transparency of reporting for systematic reviews.

### Inclusion/exclusion criteria

Studies on the psychometric properties of PAQs were only considered eligible if: 1) published in English; 2) physical activity questionnaires were developed in English; 3) administered to adults (>18yo) in English-speaking countries either with, or without pathological conditions (e.g., cardiovascular disease, musculoskeletal disease, metabolic disease or respiratory disease); 4) questionnaires consisted of recall methods of the past-week (i.e., previous 7 days) and usual-week (i.e., previous 7 days over 1–12 months); 5) questionnaires classified physical activity level based on energy expenditure, step count, distance travelled or duration of physical activity with the corresponding metabolic equivalent of task (MET); and 6) if the questionnaires were administered by trained interviewers. Studies were excluded if: 1) published as abstracts, 2) conference proceedings or dissertations; 3) used questionnaires with recall methods of less than 7 days, or recall over the previous 1–12 months that do not report average physical activity level over a 7-day period (i.e., average physical activity over the past month would be excluded whilst average 7-day physical activity over the past month would be included); 4) conducted using paediatric population or those with known cognitive impairment; 5) used questionnaires were translated into a language other than English; and 6) if the questionnaires were administered to individuals from non-English speaking backgrounds as cross-cultural validation was beyond the scope of this systematic review.

### Information sources

**Fig. 1:**
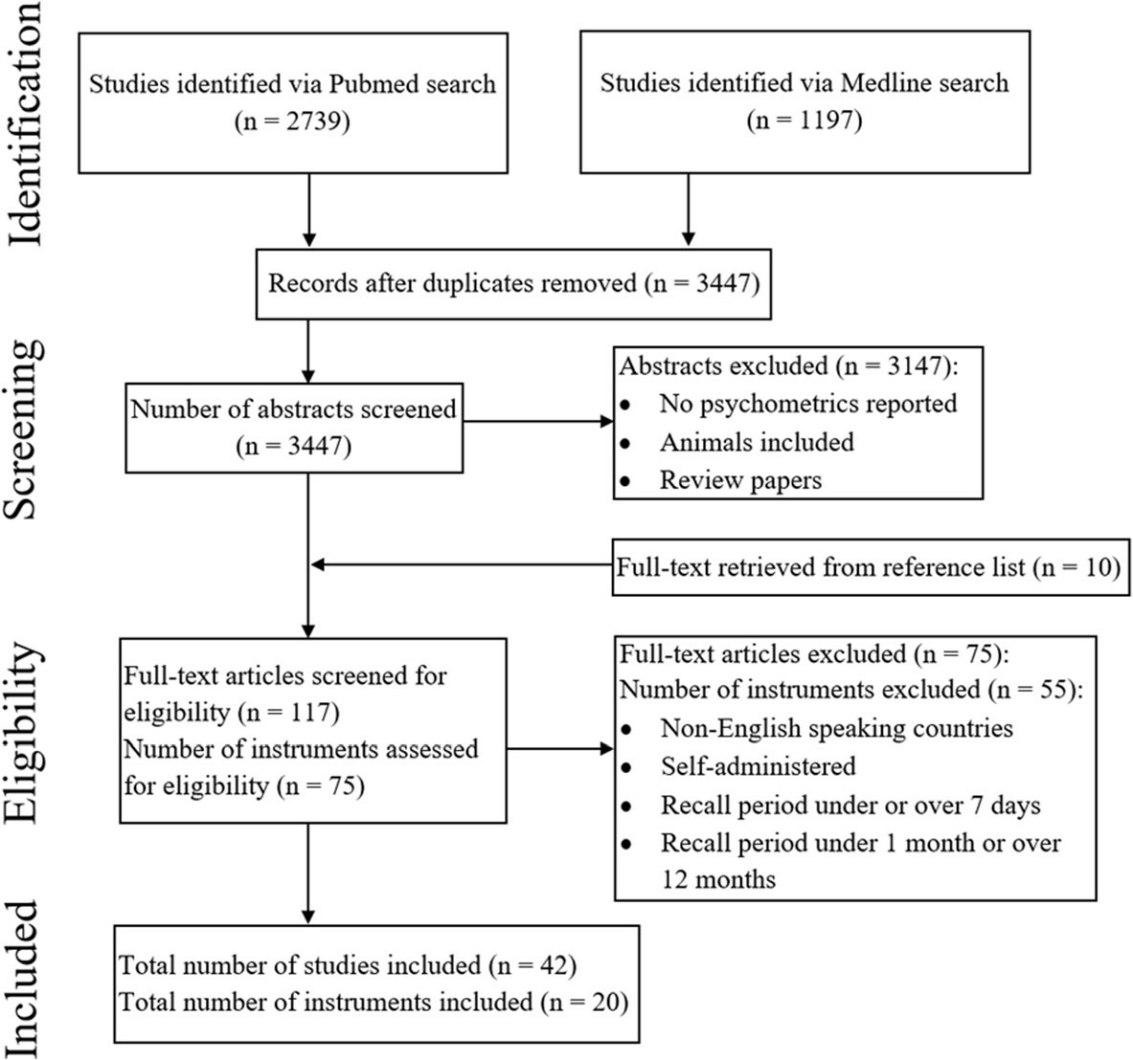
Literature search conducted based on the PRISMA guidelines

A systematic literature search was conducted by two authors in June 2017 using two electronic databases (Embase and Pubmed). Subject headings and free text were used as part of the search for both databases, with date restrictions of the past half year applied for the free text search (refer to Table 1 for all search terms used during each electronic search). Following elimination of duplicates, a total of 7191 abstracts were retrieved from the search. The search process summary in accordance with the PRISMA guidelines is depicted in Fig. 1.

**Table 1:** Search terms and databases used to obtain abstracts

Initial search: Assessment retrieval	Database and Search Terms	Limitations
Subject Headings	Embase: (Questionnaire/) AND (Physical capacity/ OR “physical constitution and health”/ OR “movement (physiology)”/ OR “physical activity, capacity and performance”/ OR Exercise/ OR Performance/ OR Motor performance/) AND (Validation study/ OR validity/ OR Psychometry/ OR Reliability/ OR Measurement accuracy/ OR measurement error/ OR measurement precision/ OR measurement repeatability/)	Humans; English; Adult: 18 to 64 years OR Aged: 65+ years
	PubMed: (“Physical Conditioning, Human”[Mesh] OR “Physical Fitness”[Mesh] OR “Physical Therapy Modalities”[Mesh] OR “Physical Endurance”[Mesh] OR “Physical Exertion”[Mesh] OR “Exercise”[Mesh] OR “Motor Activity”[Mesh] OR “Exercise”[Mesh] OR “Exercise Movement Techniques”[Mesh] OR “Exercise Therapy”[Mesh] OR “Psychomotor Performance”[Mesh] OR “Motor Skills”[Mesh] OR “Motor Activity”[Mesh]) AND (“Surveys and Questionnaires”[Mesh]) AND (“Psychometrics”[Mesh] OR “Reproducibility of Results”[Mesh] OR “Validation Studies as Topic”[Mesh] OR “Bias (Epidemiology)”[Mesh] OR “Observer Variation”[Mesh])	Humans; English; Adult: 19+ years
Free Text Words	Embase: (questionnaire*) AND (physic* OR movement* OR capacit* OR exercise* OR train* OR performance* OR motor) AND (psychometric* OR reliability OR validit* OR reproducibility OR bias)	Publication date from 2017 – current
	PubMed: *As per Embase Free Text*	Publication date from 2016/12/09 to 2017/06/09

### Selection process

Two independent reviewers with a health science background initially screened all abstracts against the pre-established inclusion/exclusion criteria, with each abstract rated as either meeting (“yes”), potentially meeting (“maybe”) or not meeting (“no”) the inclusion criteria. The reviewers were also trained by the primary author (KD) to ensure transparency of the inclusion criteria prior to abstract screening. Upon completion of abstract selection, a random 40% of abstracts were compared between the two independent reviewers and any disagreement between reviewers were consulted by a third reviewer (KD). Our calculation showed a Weighted Kappa calculation of 0.85 (95%CI: 0.81–0.90) which was considered as excellent for inter-rater reliability [26]. Original articles from selected abstracts classified as either “yes” or “maybe” were accessed for further screening by the two reviewers using the same inclusion/exclusion criteria. The first author provided final decisions if any disparity occurred between the two reviewers during the selection process of original articles.

### Assess study methodological quality using COSMIN ratings

The COSMIN taxonomy of measurement properties and definitions for health-related patient-reported outcomes were used to evaluate the methodological quality of the included studies [27] (see Table 2). The COSMIN checklist evaluates the methodological quality of studies on psychometric properties and consists of nine domains: internal consistency, reliability (test-retest reliability, inter-rater reliability and intra-rater reliability), measurement error (absolute measures), content validity, structural validity, hypothesis testing, cross-cultural validity, criterion validity and responsiveness [13]. ‘Interpretability’ is not considered to be a psychometric property; thus, it was excluded from this review. Of the nine domains, ‘responsiveness’ was not evaluated as the questionnaire’s ability to detect changes over time was beyond the scope of this review. Furthermore, ‘cross-cultural validity’ was not assessed, as questionnaires either administered in non-English speaking countries or translated into non-English languages did not meet this review’s inclusion criteria. Finally, whilst accelerometry and double-labelled water technique are considered the ‘gold standard’ of assessing physical activity level, there is a risk of under-reporting certain exercise modes (e.g., swimming or resistance training) [28]. Therefore, comparison of physical activity level reported by PAQs and objective measures (i.e., accelerometer and double-labelled water method) was considered as ‘convergent validity’.

**Table 2:** Definitions for aspects of domains and measurement properties from the COSMIN checklist by Mokkink et al. [27]

Psychometric property	Domain: Definition
	Validity: The degree to which an HR-PRO instrument measures the construct(s) it purports to measure
Content validity	The degree to which the content of an HR-PRO instrument is an adequate reflection of the construct to be measured
Face validity^a^	The degree to which an HR-PRO instrument indeed looks as though they are an adequate reflection of the construct to be measured
Construct validity	The degree to which the scores of an HR-PRO instrument are consistent with hypotheses based on the assumption that a HR-PRO instrument validly measures the construct to be measured
Structural validity^b^	The degree to which the scores of an HR-PRO instrument are an adequate reflection of the dimensionality of the construct to be measured
Hypothesis testing^b^	Item construct validity
Criterion validity	The degree to which the scores of an instrument satisfactorily reflect a “gold standard”
Responsiveness	Responsiveness: the capability of an HR-PRO instrument to detect change in the construct to be measured over time
Interpretability^c^	Interpretability: the extent to which qualitative meaning is reflective of an instrument’s quantitative scores or score change
	Reliability: The degree to which the measurement is free from measurement error
Internal consistency	The degree of the interrelatedness among the items
Reliability	The proportion of total variance in the measurements due to “true” differences amongst patients
Measurement error	The systematic and random error of a patient’s score that is not attributed to true changes in the construct to be measured

Notes: ^a^ Aspect of content validity.

^b^ Aspect of construct validity under the domain validity

^c^ Interpretability is no considered a psychometric property

Each COSMIN checklist domain consists of between 5 to 18 items which addresses various aspects of study design and statistical methods. Based on a 4-point rating system (i.e., excellent, good, fair and poor, respectively), Terwee and colleagues [13] initially suggested that the overall methodological quality of each domain should mirror the rating of the lowest-rated item (i.e., if four items were rated ‘Good’ and one ‘Poor’, the overall score would be ‘Poor’). However, given that each domain consists of items that assess a variety of methodological qualities, rating the overall methodological quality of a domain solely based on the lowest scoring single item undermines the ability of the checklist to explore subtle differences in psychometric qualities of each questionnaire [29]. Subsequently, a revised scoring method was implemented for this review by reporting the overall methodological quality of each domain as a percentage rating, as per Cordier, Speyer [29]. This revised scoring method has also been utilised successfully in a systematic review that compared Past-week and Usual-week PAQs, with sufficient sensitivity to detect differences between psychometric properties [16]. Specifically, the raw scores of each item were used to calculate a percentage of rating according to the following formula:

$$ Total\ score\ of\ each\ domain=\frac{\left(\mathrm{Total}\ \mathrm{score}\ \mathrm{obtained}-\mathrm{minimum}\ \mathrm{score}\ \mathrm{obtained}\right)}{\left(\mathrm{Highest}\ \mathrm{score}\ \mathrm{possible}-\mathrm{minimum}\ \mathrm{score}\ \mathrm{possible}\right)}\times 100 $$

The final percentage score depicting the overall methodological quality of each domain was then classified as follows: Poor = 0–25.0%, Fair = 25.1–50.0%, Good = 50.1–75.0%, Excellent = 75.1–100.0% [30]. Once the psychometric quality ratings of each paper were completed, ratings from a random 40% of papers were compared between two independent reviewers (*KD and LP*), resulting in a weighted Kappa of 0.84 (0.62–1.00), indicating excellent agreement.

### Quality of the psychometric properties

To account for varying sample sizes of each study when comparing the reliability (i.e., reproducibility) and convergent validity (a form of hypothesis testing that evaluates the correlation between two related measures, for example, physical activity levels measured from the PAQs under investigation and other measures) between PAQs, the weighted mean of correlation coefficient (i.e., *r*-values) were calculated, using the following formula:

$$ \overline{x}=\frac{\sum \limits_{i=1}^n{w}_i{x}_i}{\sum \limits_{i=1}^n{w}_i} $$

Where *w* = *r*-value of the comparison within a study (e.g., PAQ vs. another instrument or PAQ vs. Accelerometer/pedometer) and *x* = sample size of the comparison.

For the strength of reliability, once the weighted *r*-values were calculated for each study per PAQ, these measures were then averaged to compare the overall correlation between Past-week and Usual-week PAQs. For the strength of convergent validity, weighted *r*-values were averaged to compare overall correlations between Past-week and Usual-week PAQs, and between parameters that reported direct measures of PA level (e.g., diaries, other PAQs, accelerometers, pedometers) and indirect measures of PA level (e.g., aerobic fitness, muscular strength). If the sample size between each study was equivalent, then the normal non-weighted *r*-values were averaged. The strength of correlation was classified according to Cohen’s method, with the following: 0–0.29, 0.3–0.49 and ≥ 0.5 as weak, moderate and strong, respectively [31].

**Fig. 2:**
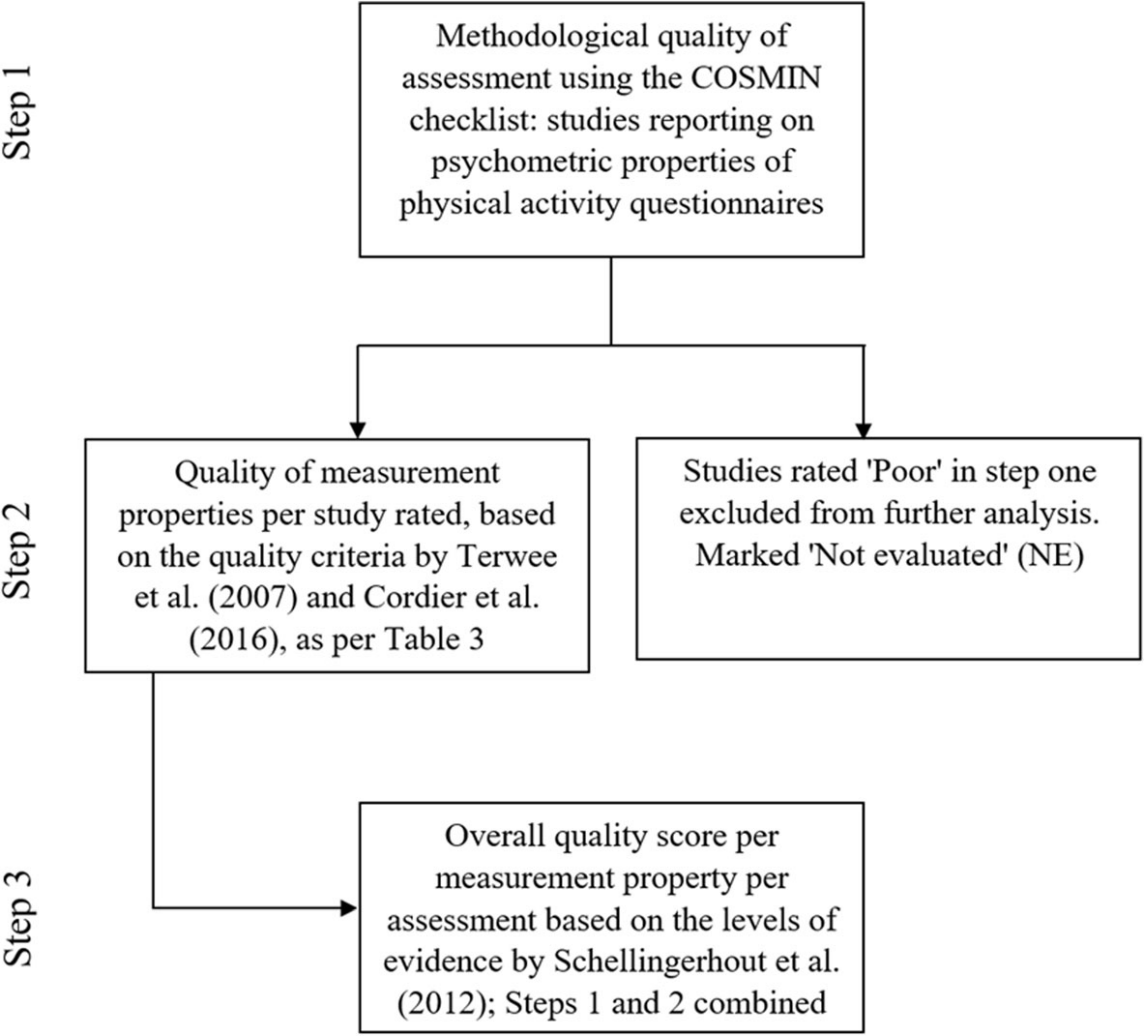
Flow chart of the methodological quality rating based on COSMIN, derivation of the quality of psychometric properties and overall quality score per measurement

The psychometric quality of each measurement property per PAQ for each study (Table 3) was also classified using the following quality criteria: “positive” (+), “conflicting” (±), “indeterminate” (?), “negative” (−), “not reported” (NR) or “not evaluated” (NE) [15, 30]. Studies that were rated as “poor” based on the COSMIN rating were excluded from further analyses and received “not evaluated” (NE). Finally, an overall quality score of assessments for each psychometric property was calculated based on the levels of evidence by Schellingerhout, Verhagen [14]. These scores were determined by integrating the methodological quality rating of the included studies on psychometric properties using the COSMIN checklist, and the quality criteria for measurement properties of assessment according to Terwee, Bot [15] and Cordier, Chen [30] (see Table 3). Figure 2 depicts a flowchart of the analysis process involved in determining the overall quality score for each assessment.

**Table 3:** The modified version of the psychometric quality rating set out by (Terwee et al., 2007) and (Cordier et al., [30])

Psychometric property	Score ^a^	Quality Criteria ^b^
Content validity	+	A clear description is provided of the measurement aim, the target population, the concepts that are being measured, and the item selection AND target population and (investigators OR experts) were involved in item selection
	?	A clear description of above-mentioned aspects is lacking OR only target population involved OR doubtful design or method
	–	No target population involvement
	±	Conflicting results
	NR	No information found on target population involvement
	NE	Not evaluated
Structural validity^c^	+	Factors should explain at least 50% of the variance
	?	Explained variance not mentioned
	–	Factors explain < 50% of the variance
	±	Conflicting results
	NR	No information found on structural validity
	NE	Not evaluated
Hypothesis testing^c^	+	Specific hypotheses were formulated AND at least 75% of the results are in accordance with these hypotheses; Convergent validity: correlation between similar assessments is at a statistically significant level (*p* < 0.05) and strength of relationship is ≥0.5 which is consistent with the hypothesis; Discriminant validity: uses appropriate statistical analysis (e.g., t-test *p* < 0.05 or Cohen’s d effect size ≥0.5)
	?	Doubtful design or method (e.g., no hypotheses)
	–	Less than 75% of hypotheses were confirmed, despite adequate design and methods; Convergent validity: correlation between similar assessments is not at a statistically significant level (*p* ≥ 0.05) and strength of relationship is < 0.5 which is inconsistent with hypothesis
	±	Conflicting results between studies within the same manual
	NR	No information found on hypotheses testing
	NE	Not evaluated
Internal consistency	+	Factor analyses performed on adequate sample size (7 * # items and 100) AND Cronbach’s alpha(s) calculated per dimension AND Cronbach’s alpha(s) between 0.70 and 0.95
	?	No factor analysis OR doubtful design or method
	–	Cronbach’s alpha(s) < 0.70 or > 0.95, despite adequate design and method
	±	Conflicting results
	NR	No information found on internal consistency
	NE	Not evaluated
Reliability	+	ICC or weighted Kappa 0.70
	?	Doubtful design or method (e.g., time interval not mentioned)
	–	ICC or weighted Kappa < 0.70, despite adequate design and method
	±	Conflicting results
	NR	No information found on reliability
	NE	Not evaluated
Measurement error^d^	+	MIC < SDC OR MIC outside the LOA OR convincing arguments that agreement is acceptable
	?	Doubtful design or method OR (MIC not defined AND no convincing arguments that agreement is acceptable)
	–	MIC SDC OR MIC equals or inside LOA, despite adequate design and method
	±	Conflicting results
	NR	No information found on measurement error
	NE	Not evaluated

*Notes*. ^a^Scores: + = positive rating,? = indeterminate rating, — = negative rating, ± = conflicting data, NR = not reported, NE = not evaluated (for study of poor methodological quality according to COSMIN rating, data are excluded from further evaluation

^b^Doubtful design or method is assigned when a clear description of the design or methods of the study is lacking, sample size smaller than 50 subjects (should be at least 50 in every subgroup analysis), or any important methodological weakness in the design or execution of the study

^c^Hypothesis testing: all correlations should be statistically significant (if not, these hypotheses are not confirmed) AND these correlations should be at least moderate (*r* > 0.5)

^d^Measurement error: *MIC* minimal important change, *SDC* smallest detectable change, *LOA* limits of agreement

### Data items and synthesis of results

Domains from the COSMIN checklist and psychometric property qualities were assessed for each included study according to Terwee, Bot [15] and Cordier, Chen [30]. The results were then reported in the following order: 1) the description of the literature search (see Table 1); 2) the characteristics of the interview-based PAQ measures (see Table 4) and studies reporting on the development and validation of the interview-based PAQ measures (see Table 4); 3) the methodological quality according to the COSMIN checklist of each study that have reported on the psychometric properties of PAQs (see Table 6); 4) the comparisons of the average weighted *r*-values of test-retest reliability and convergent validity between Past-week and Usual-week PAQs (see Table 7); 5) the quality of relevant psychometric properties for each study based on the criteria by Terwee, Bot [15] and Cordier, Chen [30] (Table 8); and 6) the overall quality rating of psychometric properties based on the levels of evidence by Schellingerhout, Verhagen [14] for each PAQ and comparing these results between Past-week and Usual-week PAQs (see Table 9).

**Table 4:** Characteristics of interview-administered Past-week and Usual-week physical activity questionnaires

Instrument	Purpose of instrument	Published year	Type of administration/Recall method	Number of subscales/forms	Total number of items	Response options
Usual-week Physical Activity Questionnaires						
CaMos *Usual-week*	To assess physical activity among those with susceptibility to osteoporosis	2004	Usual 7-days over 12 months	4	10	Type of occupation: • Full-time/Part-time/Unemployed/Disabled/Retired • Mostly sitting/Mostly standing or walking/Usually lift light loads/Usually lift heavy loads Strenuousness of activity: • Hours/week Sitting activities: • Hours/week Sleep: • Hours/day
IPEQ-WA *Usual-week*	To assess incidental physical activity among older adults	2010	Usual 7-days over 3 months	10	16	Type of activity: • Minutes/week Walking for exercise: • Times/week • Minutes/bout Incidental walking: • Times/week • Minutes/bout House maintenance/gardening: • Minutes/day Time on feet indoors: • Minutes/day
MAQ *Usual-week*	To assess physical activity among the general population	1990	Usual 7-days over 12 months	6	9	Type of activity: • Times/month • Minutes/bout Television viewing: • Hours/day Confined to bed or chair from injury/illness: • Weeks/year Difficulties with activities: • Yes/no Types of sports: • Total years Walk/cycle to work: • Min/day
NHS II *Usual 7-days*	To assess the health conditions of nurses working at hospitals	1989	Usual 7-days over 12 months	3	16	Daily flights of stairs: • ≤ 2; 3–4; 5–9; 10–14; ≥ 15 Physical activity per week: • 0–11+ hours Sedentary time per week: • 0–90+ hours
Phone-FITT *Usual-week*	To assess physical activity among older adults via phone	2008	Usual 7-days over 1 month	9	16	Type of household activity: • Hours/week Type of recreational activity: • Hours/week Type of seasonal recreational activity: • Hours/week Other physical activity: • Hours/week
YPAS *Usual 7-days*	To assess physical activity among Volder adults	1993	Usual 7-days over 1 month	6	39	Type of activity: • Hours/week Vigorousness of activity: • Frequency /week or /month Leisurely walk: • Frequency /week or /month; Duration in minutes General movement: • Hours/day Standing and sitting: • Hours/day Seasonal changes: • Compare current season
Past-week Physical Activity Questionnaires						
AAS *Past 7-days*	Population surveillance of physical activity in Australian adults	2003	Past 7-days	4	8	Walking activities: • Frequency/week; Hours/week; minutes/week Vigorous yard work: • Frequency/week; Hours/week; minutes/week Vigorous activities other than yard work: • Frequency/week; Hours/week; minutes/week Moderate activities: • Frequency/week; Hours/week; minutes/week
AAS (modified)	Population surveillance of physical activity in Australian adults	2013	Past 7-days	4	8	Walking activities: • Frequency/week; Hours/week; minutes/week Vigorous yard work: • Frequency/week; Hours/week; minutes/week Vigorous activities other than yard work: • Frequency/week; Hours/week; minutes/week Moderate activities: • Frequency/week; Hours/week; minutes/week
CAQ-PAI *Past 7-days*	To measure overall kilocalories expended in leisure-time physical activity	1978	Past 7-days	3	4	Walking: • Blocks/day Stairs: • Flights/day Recreational activities: • Frequency/week; Hours/week; minutes/session
Checklist Questionnaire *Past 7-days*	Assess the frequency and duration of physical activities performed in the previous 7 days	2012	Past 7-days	10	64	Household activities: • Frequency/week; Hours/week; minutes/week Yard activities: • Frequency/week; Hours/week; minutes/week Family activities: • Frequency/week; Hours/week; minutes/week Community/volunteer/church: • Frequency/week; Hours/week; minutes/week Transportation: • Frequency/week; Hours/week; minutes/week Miscellaneous: • Frequency/week; Hours/week; minutes/week Other time: Exercise, sports and dancing: • Frequency/week; Hours/week; minutes/week Employment: • Frequency/week; Hours/week; minutes/week Miscellaneous: • Frequency/week; Hours/week; minutes/week
Global Questionnaire	To assess physical activity among older adults	2001	Past 7-days	5	35	Type of activity: • Hours/week Vigorousness of activity: • Frequency/week or /month Leisurely walk: • Frequency/week or /month; Duration in minutes General movement: • Hours/day
IPAQ-LF *Past 7-days*	As for IPAQ-LF (Telephone)	2002	Past 7-days	As for IPAQ-LF (Telephone)	As for IPAQ-LF (Telephone)	Vigorous activities • Days/week; Hours/week; minutes/day Moderate activities • Days/week; Hours/week; minutes/day Light activities • Days/week; Hours/week; minutes/day Sitting time • Days/week; Hours/week; minutes/day
IPAQ-SF *Past 7-days*	As for IPAQ-LF (Telephone version)	2002	Past 7-days	4	7	Vigorous activities • Days/week; Hours/week; minutes/day Moderate activities • Days/week; Hours/week; minutes/day Light activities • Days/week; Hours/week; minutes/day Sitting time • Days/week; Hours/week; minutes/day
NZPAQ-LF	Retrospective diary that assesses all dimensions of physical activity type and level in New Zealand	2008	Past 7-days	5	11	Sport/recreation: • Days/week; Hours/week; minutes/day Transport: • Days/week; Hours/week; minutes/day Occupation: • Days/week; Hours/week; minutes/day Cultural/incidental activities: • Days/week; Hours/week; minutes/day Inactivity: • Days/week; Hours/week; minutes/day
NZPAQ-SF	Modified version of IPAQ-SF to assess physical activity level whilst reflecting the culture in New Zealand	2008	Past 7-days	4	7	Walking activities: • Days/week; Hours/week; minutes/day Moderate physical activity: • Days/week; Hours/week; minutes/day Vigorous physical activity • Days/week; Hours/week; minutes/day: Frequency of activity: • Days/week; Hours/week; minutes/day
PAAQ	To assess physical activity level in line with the Canadian Physical Activity Guidelines	2015	Past 7-days	4	12	Walking or cycling to destination • Yes/No • Days/week; Hours/week; minutes/day Recreational activities, organised activities lasting minimum of 10 min that caused sweat or hard breathing: • Yes/No • Days/week; Hours/week; minutes/day Activities at work, home or volunteering that caused sweat or hard breathing: • Yes/No • Days/week; Hours/week; minutes/day Vigorous exercises that caused breathlessness: • Yes/No • Days/week; Hours/week; minutes/day
PASE *Past 7-days*	To assess leisure, occupational and household physical activities amongst the elderly	1991	Past 7-days	3	27	Recreational activities: • Frequency/week; < 1 h, 1–2 h, 2–4 h or > 4 h Household activities: • Yes or no; Type of activities Occupational activities: • Hours/week; Type of activities
PWMAQ	To assess leisure physical activities during the past week	2009	Past 7-days	6	9	Type of activity: • Times/week • Minutes/bout Television viewing: • Hours/day Confined to bed or chair from injury/illness: • Minutes/week Difficulties with activities: • Yes/no Types of sports: • Total years Walk/cycle to work: • Min/day
PAR *Past 7-days*	To assess sleep and physical activity patterns	1985	Past 7-days	6	15	Occupational activities: • Yes or No; Frequency/week; Hours/week; Days/week Moderate, Hard and Very Hard in the Morning: • Minutes Moderate, Hard and Very Hard in the Afternoon: • Minutes Moderate, Hard and Very Hard in the Evening: Strength: • Minutes Flexibility: • Minutes
VAPAQ *Past 7-days*	To measures physical activities amongst veterans	2003	Past 7-days	3	6	Walking activities: • Blocks/day Sports/recreational activities: • Frequency/week; Hours/week; minutes/session Occupational activities: • Frequency/week; Hours/week; minutes/session

*CaMos* Canadian Multicentre Osteoporosis Study, *IPEQ-WA* Incidental and Planned Exercise Questionnaire, *MAQ* Modified Activity Questionnaire, *NHS II* Nurses’ Health Study version II, *Phone-FITT* Phone Fitness, *YPAS* Yale Physical Activity Survey, *AAS* Active Australia Survey, *CAQ-PAI* College Alumni Questionnaire – Physical Activity Index, *IPAQ-LF* International Physical Activity Questionnaire Long Form, *IPAQ-SF* International Physical Activity Questionnaire Short Form, *NZPAQ-LF* New Zealand Physical Activity Questionnaire Long Form, *NZPAQ-SF* New Zealand Physical Activity Questionnaire Short Form, *PAAQ* Physical Activity Adult Questionnaire, *PASE* Physical Activity Scale for the Elderly, *PWMAQ* Past Week Modified Activity Questionnaire, *PAR* Physical Activity Recall Questionnaire, *VAPAQ* Veterans Physical Activity Questionnaire

## Results

### Systematic literature search

Following removal of duplicate abstracts, a total of 3447 abstracts were screened according to the inclusion criteria. Upon completion of screening, 75 PAQs and 117 of their corresponding full-text articles were examined for eligibility. Amongst these items, 20 PAQs and 42 of their corresponding articles were included. The remaining 55 PAQs were excluded for the following reasons: non-specified recall periods; recall period was beyond 7 days; recall period was less than 7 days; and various combinations of recall periods.

### Included physical activity questionnaires

Table 4 displays the characteristics of the included PAQs, with description of their corresponding studies shown in Table 5. There were 6 PAQs that assessed Usual 7-days of PA level with two PAQs that had a 1-month recall period (Phone FITT and YPAS), one PAQ that had a 3-month recall period (IPEQ-WA) and three PAQs that had a 12-month recall period (CaMos, MAQ and NHS II; Table 4). The remaining 13 PAQs encompassed items that assessed PA level over the Past-7 days. A majority of PAQs had subscales that were separated by the intensity of PA (e.g., light, moderate and vigorous), whereas other PAQs had subscales categorised by the mode of PA (e.g., walking, stairs, occupational and gardening activities).

**Table 5:** Description of studies for the development and validation of interview-administered Past-week and Usual-week physical activity questionnaires

Instrument	Reference	Purpose of study	Study population	Health condition	Age range (R; mean ± standard deviation)
Usual-week Physical Activity Questionnaires					
CaMos *Usual-week*	Nadalin, Bentvelsen [45]	To assess test-retest reliability of a portion of the CaMos questionnaire using a combination of administration modes	Reliability (*N* = 367)	*Physical*: healthy with possible osteoporosis *Cognitive*: Not screened	*Total sample*: R = 45–80 (NR)y
IPEQ-WA *Usual-week*	Merom, Delbaere [46]	Assessed construct validity and responsiveness of IPEQ	*Male (I) & Female (II)*: Validity (*N* = 40 & 86)	*Physical*: No chronic disease conditions *Cognitive*: Healthy based on cognitive test	*Total sample*: R = NR; *(I)* NR; *(II)* NR
MAQ *Usual-week*	Pettee Gabriel, McClain [47]	Test-retest reliability and convergent validity of five PAQs commonly used in larger health studies involving middle-aged women	*Female (I)*: Repeatability & Validity (*N* = 62–66)	*Physical*: No chronic disease conditions *Cognitive*: Not screened	*Total sample*: R = 45–65 (52.6 ± 5.4)y
	Kriska, Knowler [48]	To examine the reliability and validity of the MAQ	*Male (I) & Female (II*): Repeatability (*N* = 69) Validity (*N* = 21)	*Physical*: No physical limitations with possible type II diabetes mellitus *Cognitive*: Not screened	*Total sample:* R = 10–59 (NR)yr.; *(I)* 10–59 NR; *(II)* 10–59 (NR)y
	Kriska, Edelstein [49]	To compare MAQ with other PAQs among individuals with type 2 diabetes	*Male:* Validity *(I)* (*N* = 1043) *Female:* Validity *(II)* (*N* = 2191)	*Physical*: No physical limitations with possible type II diabetes mellitus *Cognitive*: Not screened	*Total sample:* NR (50.6 ± 10.7)yr.; *(I)* NR; *(II)* NR
	Schulz, Harper [50]	To compare MAQ with direct measures of energy expenditure	*Male:* Validity *(I)* (*N* = 12) *Female:* Validity *(II)* (N = 9)	*Physical*: No physical limitations with possible type II diabetes mellitus *Cognitive*: Not screened	*Total sample:* NR; *(I)* R = NR (35.4 ± 13.8)yr.; *(II)* R = NR (31.3 ± 13.0)y
NHS II *Usual-week*	Pettee Gabriel, McClain [47]	As for MAQ	As for MAQ	As for MAQ	As for MAQ
Phone-FITT *Usual-week*	Gill, Jones [51]	To develop the Phone-FITT and to evaluate the test–retest reliability and criterion-related (concurrent) and construct (convergent, discriminant and known-groups) validity	*Male*: Repeatability *(I)* & Validity *(II)* (*N* = 22 & 12) *Female*: Repeatability *(III)* & Validity *(IV)* (N = 21 & 36)	*Physical*: No chronic disease conditions *Cognitive*: Not screened	*Total sample*: R = 73–87 (79.4 ± 2.9)y; *(I)* 76–86 (79.4 ± 3.2)y; *(II)* 72–82 (76.5 ± 3.4); *(III)* 76–86 (79.5 ± 2.7)y; *(IV)* 71–89 (77.8 ± 5.1)y
YPAS *Usual-week*	Colbert, Matthews [52]	Compared validity of a variety of physical activity measurement tools in older adults	Validity (*N* = 56)	*Physical*: Musculoskeletal conditions, lung disease, cancer and hypertension *Cognitive*: Not screened	*Total sample*: R = NR (74.7 ± 6.5)y
	Dipietro, Caspersen [53]	Preliminary repeatability data and validation results relative to selected physiologic variables	*Male (I) & Female (II)*: Repeatability (*N* = 20 & 56); Validity (*N* = 14 & 11)	*Physical*: No chronic disease conditions *Cognitive*: Not screened	*Total sample*: R = NR (71.0 ± 6.6)y; *(I)* R = NR (70.9 ± 6.2)y; *(II)* R = NR (69.6 ± 6.0)y
	Gennuso, Matthews [54]	Reliability and validity of physical activity surveys for assessing time spent in sedentary behavior in older adults	Validity & Repeatability (*N* = 58)	*Physical*: NR *Cognitive*: Not screened	*Total sample*: R = 66–88 (75.1 ± 6.5)y
	Harada, Chiu [55]	Assess the known-groups and construct validity of CHAMPS, PASE and YPAS	*Retirement homes (I) & Community centres (II)*: Validity (N = 36 & 51)	*Physical*: Musculoskeletal conditions, lung disease, diabetes and hypertension *Cognitive*: Healthy based on cognitive test	*Total sample*: R = 56–89 (75.0 ± 6.0); *(I)* R = 65–89 (79.0 ± 6.0); *(II)* R = 65–86 (73.0 ± 5.0)
	Kolbe-Alexander, Lambert [56]	Validity and reliability of the YPAS and the short version of the IPAQ in older South African adults	*Male (I) & Female (II)*: (*N* = 52 & 70); Sample (N) not reported between psychometric measures	*Physical*: NR *Cognitive*: Not screened	*Total sample*: R = 62–69 (66 ± NR)y; *(I)* 62–69 (67 ± NR); *(II)* 62–69 (65 ± NR)
	Moore, Ellis [57]	Construct validity of four PAQs in culturally diverse older adults	*African American (I) & Caucasian (II)*: Validity (*N* = 54)	*Physical*: Musculoskeletal conditions, neurological and cardiorespiratory *Cognitive*: Healthy based on cognitive test	*Total sample*: NR; *(I)* NR (67.2 ± 9.9)y; *(II)* NR (66.3 ± 9.8)y
Past-week Physical Activity Questionnaires					
AAS *Past-7 days*	Brown, Trost [58]	Assessed the test-retest reliability of activity status derived from four physical activity measures	*AAS (I), IPAQ (II), BRFSS (III) & NHS (IV)*: Repeatability (*N* = 356, 104, 127 & 122)	*Physical*: NR *Cognitive*: Not screened	*Total sample*: R = 18-75y (NR)y; *(I), (II), (III) & (IV)* 18-75y (NR)y
	Brown, Bauman [59]	Compared the level of agreement in prevalence estimates of the proportion of the population that is sufficiently active for health benefit derived from four measures that are in use in Australia and elsewhere around the world	*AAS (I), IPAQ (II) & BRFSS (III)*: Validity (*N* = 428, 427 & 425)	*Physical*: NR *Cognitive*: Not screened	*Total sample*: R = 18-75y (NR)y; *(I), (II) & (III)* 18-75y (NR)y
	Creamer, Bowles [60]	Determining computer-assisted approaches for surveillance of physical activity	Validity & Repeatability (N = 56)	*Physical*: NR *Cognitive*: Screened based on capability to read	*Total sample*: NR (43.1 ± 11.4)y
AAS (modified) *Past-7 days*	Fjeldsoe, Winkler [18]	Determined the test–retest reliability and criterion validity the Adapted Active Australia Survey and whether these properties varied across participants’ activity levels	Validity & Repeatability (N = 63)	*Physical*: NR *Cognitive*: Screened based on capability to read	*Total sample*: NR (49.5 ± 12.5)y
CAQ-PAI *Past-7 days*	Mahabir, Baer [61]	Convergent validity of four physical activity questionnaires with DLW	Validity (*N* = 65)	*Physical*: No chronic disease conditions *Cognitive*: Not screened	*Total sample*: 49.2–78.8 (59.9 ± 7.5)y
	Rauh, Hovell [62]	Reliability and convergent validity of several PAQs	Validity (*N* = 45)	*Physical*: NR *Cognitive*: Not screened	*Total sample:* 18–55 (33.0 ± 10.6)y
	Washburn, Smith [63]	Reliability of the CAQ-PAI	*Combined gender (I), Male (II) & Female (III)*: Repeatability (*N* = 633, 261 & 372)	*Physical*: No chronic disease conditions *Cognitive*: Not screened	*(I) Total sample*: 25–65: (39.5 ± 10.8)y; *(II)* NR (38.2 ± 10.6)y; *(III)* NR (40.5 ± 10.8)
Checklist Questionnaire *Past-7 days*	Masse, Fulton [64]	Compared the validity of two physical activity questionnaire formats	Validity (*N* = 260)	*Physical*: NR *Cognitive*: Not screened	*Total sample*: R = 40–70 (49.2 ± 7.0)y
Global Questionnaire *Past-7 days*	Masse, Fulton [64]	As per Checklist Questionnaire	As per Checklist Questionnaire	As per Checklist Questionnaire	As per Checklist Questionnaire
IPAQ-LF *Past-7 days*	Ahn, Chmiel [65]	Validity of IPAQ-SF (telephone) with accelerometer amongst adults with systemic lumpus erythematosus	Validity (*N* = 118)	*Physical*: Systemic Lupus Erythematosus *Cognitive*: Not screened	*Total sample*: NR (45.4 ± 10.9)y
	Garriguet, Tremblay [66]	Validity of IPAQ-LF (self-administered) and the new Physical Activity for Adults Questionnaire (PAAQ) with accelerometers	*IPAQ-LF (I) & PAAQ (II)*: Validity (*N* = 94 & 108)	*Physical*: NR *Cognitive*: Healthy based on cognitive interview	*Total sample*: 18–79 (NR); *(I)* NR (47 ± NR)y; *(II)* NR (47 ± NR)y
IPAQ-SF *Past-7 days*	Ainsworth, Macera [67]	Compared the physical activity prevalence estimates obtained from BRFSS and IPAQ-SF (interview)	Validity (*N* = 9945)	*Physical*: Non-institutionalised *Cognitive*: Non-institutionalised	*Total sample*: R = 18–55+ (NR)
	Brown, Trost [58]	As for AAS	As for AAS	As for AAS	As for AAS
	Brown, Bauman [59]	As for AAS	As for AAS	As for AAS	As for AAS
NZPAQ-LF *Past-7 days*	Moy, Scragg [68]	Convergent validity of NZPAQ-LF with heart-rate monitoring	*Male (I) & Female (II)*: Validity (*N* = 90 & 96)	*Physical*: NR *Cognitive*: Not screened	*Total sample*: 19–86 (48.6 ± 16.4)y; *(I)* NR *(*48.4 ± NR)y; *(II)* NR (48.7 ± NR)y
NZPAQ-SF *Past-7 days*	Moy, Scragg [68]	As per NZPAQ-LF	As per NZPAQ-LF	As per NZPAQ-LF	As per NZPAQ-LF
PAAQ *Past-7 days*	Garriguet, Tremblay [66]	As for IPAQ-LF	As for IPAQ-LF	As for IPAQ-LF	As for IPAQ-LF
PASE *Past-7 days*	Colbert, Matthews [52]	As for YPAS	As for YPAS	As for YPAS	As for YPAS
	Dinger, Oman [69]	Convergent validity and reliability of PASE with accelerometers with elderly individuals	Validity & Repeatability (N = 56)	*Physical*: NR *Cognitive*: Not screened	*Total sample*: NR (75.7 ± 7.9)y
	Johansen, Painter [70]	Convergent validity of three physical activity questionnaires with accelerometers in patients with end-stage renal disease	Validity (*N* = 39)	*Physical*: Patients undergoing haemodialysis *Cognitive*: Not screened	*Total sample*: NR (52 ± 16)y
	Moore, Ellis [57]	As for YPAS	As for YPAS	As for YPAS	As for YPAS
	Washburn, Smith [71]	Convergent validity and reliability of PASE with accelerometers	Validity & Repeatability (*N* = 119)	*Physical*: Included participants without serious physical impairments *Cognitive*: Included participants without serious cognitive impairments, but screening method not clear	*Total sample*: NR (73.4 ± NR)y
PWMAQ *Past-7 days*	Pettee Gabriel, McClain [72]	Reliability and validity of PWMAQ in middle-aged women	Validity & Repeatability (*N* = 66)	*Physical*: NR *Cognitive*: Not screened	*Total sample*: NR (52.6 ± 5.4)y
	Pettee Gabriel, McClain [47]	As for MAQ	As for MAQ	As for MAQ	As for MAQ
PAR *Past-7 days*	Albanes, Conway [73]	As for CAQ-PAI	As for CAQ-PAI	As for CAQ-PAI	As for CAQ-PAI
	Blair, Haskell [74]	Construct validity of PAR	*Male (I) & Female (II)*: Validity (*N* = 1077, 1206)	*Physical*: NR *Cognitive*: Not screened	*Total sample*: 16–74 (NR)y
	Conway, Seale [75]	Convergent validity of PAR with DLW	Validity (*N* = 24)	*Physical*: No chronic disease conditions *Cognitive*: Not screened	*Total sample*: 27–65 (41.2 ± 2.0)y
	Garfield, Canavan [76]	As for PASE	As for PASE	As for PASE	As for PASE
	Gross, Sallis [77]	Inter-rater reliability of PAR	Inter-rater reliability (N = 21)	*Physical*: NR *Cognitive*: Not screened	*Total sample*: 19–52 (NR)y
	Irwin, Ainsworth [78]	Convergent validity of PAR with DLW	Validity (*N* = 24)	*Physical*: No chronic disease conditions *Cognitive*: Not screened	*Total sample*: 27–65 (41.2 ± 9.6)y
	Johansen, Painter [70]	As for PASE	As for PASE	As for PASE	As for PASE
	Mahabir, Baer [61]	As for CAQ-PAI	As for CAQ-PAI	As for CAQ-PAI	As for CAQ-PAI
	Rauh, Hovell [62]	As for CAQ-PAI	As for CAQ-PAI	As for CAQ-PAI	As for CAQ-PAI
	Sallis, Haskell [79]	Reliability of PAR	Repeatability (*N* = 64)	*Physical*: NR *Cognitive*: Not screened	*Total sample*: 20–74 (40.1 ± 15.7)y
	Sarkin, Johnson [33]	Construct validity of three physical activity questionnaires	*Combined gender (I), Male (II) & Female (III)*: Validity (*N* = 575, 256 & 319)	*Physical*: NR *Cognitive*: Not screened	*(I) Total sample*: NR (24.5 ± 1.9)y; *(II)* NR (24.7 ± 2.0)y; *(III)* NR (24.4 ± 2.1)y
	Taylor, Coffey [80]	Convergent validity of PAR with motion sensors	Validity (*N* = 30)	*Physical*: Some patients with myocardial infarction several 11–26 weeks prior to study *Cognitive*: Not screened	*Total sample*: 34–69 (52.3 ± NR)
	Washburn, Jacobsen [81]	Convergent validity of PAR with DLW	*Male (I) & Female (II)*: Validity (*N* = 17 & 29)	*Physical*: No chronic disease conditions *Cognitive*: Not screened	*Total sample*: 17–35 (23.6 ± 4.2)y; *(I)* NR (23.9 ± 3.8)y; *(II)* NR (23.3 ± 4.6)y
	Williams, Klesges [82]	Reliability and convergent validity of PAR in college students	Repeatability & Validity (N = 45)	*Physical*: NR *Cognitive*: Not screened, but were all enrolled at a university	*Total sample:* 18–52 (24.7 ± 7.73)y
VAPAQ *Past-7 days*	Betz, Myers [83]	Reproducibility of VAPAQ in an elderly population	*Exercise group (I) & Usual care group (II)*: Repeatability (*N* = 26 & 29)y	*Physical*: All patients had abdominal aortic aneurysm *Cognitive*: Not screened	*Total sample*: NR (73.0 ± 7.9)y; *(I)* NR; *(II)* NR

*CaMos* Canadian Multicentre Osteoporosis Study, *IPEQ-WA* Incidental and Planned Exercise Questionnaire, *MAQ* Modified Activity Questionnaire, *NHS II* Nurses’ Health Study version II, *Phone-FITT* Phone Fitness, *YPAS* Yale Physical Activity Survey, *AAS* Active Australia Survey, *CAQ-PAI* College Alumni Questionnaire – Physical Activity Index, *IPAQ-LF* International Physical Activity Questionnaire Long Form, *IPAQ-SF* International Physical Activity Questionnaire Short Form, *NZPAQ-LF* New Zealand Physical Activity Questionnaire Long Form, *NZPAQ-SF* New Zealand Physical Activity Questionnaire Short Form, *PAAQ* Physical Activity Adult Questionnaire, *PASE* Physical Activity Scale for the Elderly, *PWMAQ* Past Week Modified Activity Questionnaire, *PAR* Physical Activity Recall Questionnaire, *VAPAQ* Veterans Physical Activity Questionnaire

### Psychometric properties of PAQs

Table 6 provides an overview of the methodological quality assessment of studies reporting on psychometric properties of usual-week and past-week physical activity questionnaires using the COSMIN checklist. The most frequently reported psychometric properties based on the COSMIN rating assessment was *hypothesis testing* (18 of 20 PAQs), ranging from fair to excellent qualities, followed by *reliability* (13 of 20 PAQs), ranging from good to excellent qualities. The least reported psychometric properties included *measurement error* (4 of 20 PAQs), ranging from good to excellent qualities, *internal consistency* (3 of 20 PAQs), ranging from poor to fair qualities and *content validity* (3 of 20 PAQs), ranging from fair to good qualities. No studies were identified that reported *structural validity*. When different PAQ recall methods were compared (i.e., Past-week PAQ versus Usual-week PAQ), similar frequencies in psychometric properties were found for Usual 7-day PAQs and Past 7-day PAQs with *internal consistency* (16.7 and 14.3%, respectively) and *content validity* (16.7 and 14.3%, respectively). However, notable differences were also shown with Usual 7-day PAQs more frequently reported for *reliability* (83.3% vs. 57.1%) and Past 7-day PAQs more frequently for *measurement error* (24.1% vs. 16.7%) and *hypothesis testing* (92.9% vs. 83.3%).

**Table 6:** Overview of the methodological quality assessment of studies reporting on psychometric properties of interview-administered Usual-week and Past-week physical activity questionnaires using the COSMIN checklist

Instrument	Study	Measurement properties					
		Internal consistency	Reliability	Measurement error	Content validity	Structural validity	Hypothesis testing^ab^
							*Type: Score*
Usual-week Physical Activity Questionnaires							
CaMos *Usual-week*	Nadalin, Bentvelsen [36]	NR	78.1% (Excellent)^c^	NR	NR	NR	NR
IPEQ *Usual-week*	Merom, Delbaere [37]	NR	NR	NR	NR	NR	Direct: 68.7% (Good) Indirect: 43.5% (Fair)
MAQ *Usual-week*	Pettee Gabriel, McClain [38]	NR	71.4% (Good)^c^	NR	NR	NR	Direct: 69.6% (Good) Indirect: 59.1% (Good)
	Kriska, Knowler [39]	NR	62.1% (Good)^c^	NR	NR	NR	Direct: 43.5% (Fair)
	Kriska, Edelstein [40]	NR	NR	NR	NR	NR	Direct: 78.3% (Excellent)
	Schulz, Harper [41]	NR	NR	NR	NR	NR	Direct: 34.8% (Fair)
NHS II *Usual-week*	Pettee Gabriel, McClain [38]	NR	71.4% (Good)^c^	NR	NR	NR	Direct: 69.6% (Good) Indirect: 59.1% (Good)
Phone-FITT *Usual-week*	Gill, Jones [32]	NR	62.1% (Good)^c^	NR	28.5% (Fair)	NR	Direct: 69.6% (Good) Indirect: 68.1% (Good)
YPAS *Usual-week*	Colbert, Matthews [42]	NR	72.4% (Good)^c^	75.9% (Excellent)	NR	NR	Direct: 82.4% (Excellent)
	Dipietro, Caspersen [43]	21.7% (Poor)	82.8% (Excellent)^†^	65.5% (Good)	NR	NR	Direct: 56.5% (Good)
	Gennuso, Matthews [44]	NR	71.9% (Good)^c^	NR	NR	NR	Direct: 60.9% (Good)
	Harada, Chiu [45]	NR	NR	NR	NR	NR	Direct: 68.1% (Good) Indirect: 75.5% (Excellent)
	Kolbe-Alexander, Lambert [46]	NR	56.3% (Good)^c^	58.6% (Good)	NR	NR	Direct: 67.4% (Good)
	Moore, Ellis [47]	NR	NR	NR	NR	NR	Indirect: 73.9% (Good)
Past-week Physical Activity Questionnaires							
AAS *Past-7 days*	Brown, Bauman [49]	NR	78.1% (Excellent)^c^	NR	NR	NR	NR
	Brown, Trost [48]	NR	NR	NR	NR	NR	Direct: 60.9% (Good)
	Creamer, Bowles [50]	NR	72.4% (Good)^c^	NR	NR	NR	Direct: 82.6% (Excellent)
AAS (modified) *Past-7 days*	Fjeldsoe, Winkler [12]	NR	72.2% (Good)^c^	72.4% (Good)	NR	NR	Direct: 78.3% (Excellent)
CAQ-PAI *Past-7 days*	Mahabir, Baer [51]	NR	NR	NR	NR	NR	Direct: 54.3% (Good)
	Rauh, Hovell [52]	NR	65.5% (Good)^c^	NR	NR	NR	Direct: 60.9% (Good) Indirect: 60.9% (Good)
	Washburn, Smith [53]	NR	72.4% (Good)^c^	NR	NR	NR	Indirect: 65.2% (Good)
Checklist Questionnaire *Past-7 days*	Masse, Fulton [54]	NR	NR	NR	NR	NR	Direct: 69.6% (Good)
Global Questionnaire *Past-7 days*	Masse, Fulton [54]	NR	NR	NR	NR	NR	Direct: 69.6% (Good)
IPAQ-LF *Past-7 days*	Ahn, Chmiel [55]	NR	NR	NR	NR	NR	Direct: 78.3% (Excellent)
	Garriguet, Tremblay [56]	NR	NR	NR	NR	NR	Direct: 69.6% (Good)
IPAQ-SF *Past-7 days*	Ainsworth, Macera [57]	NR	NR	NR	NR	NR	Direct: 78.2% (Excellent)
	Brown, Bauman [49]	NR	78.1% (Excellent)^c^	NR	NR	NR	NR
	Brown, Trost [48]	NR	NR	NR	NR	NR	Direct: 60.9% (Good)
NZPAQ-LF *Past-7 days*	Moy, Scragg [58]	NR	NR	NR	NR	NR	Indirect: 82.6% (Excellent)
NZPAQ-SF *Past-7 days*	Moy, Scragg [58]	NR	NR	NR	NR	NR	Indirect: 82.6% (Excellent)
PAAQ *Past-7 days*	Garriguet, Tremblay [56]	NR	NR	NR	57.1% (Good)	NR	Direct: 78.3% (Excellent)
PASE *Past-7 days*	Colbert, Matthews [42]	NR	72.4% (Good)^c^	75.9% (Excellent)	NR	NR	Direct: 82.4% (Excellent)
	Dinger, Oman [59]	NR	72.4% (Good)^c^	NR	NR	NR	Direct: 82.6% (Excellent)
	Johansen, Painter [60]	NR	NR	NR	NR	NR	Direct: 69.6% (Good) Indirect: 43.5% (Fair)
	Moore, Ellis [47]	NR	NR	NR	NR	NR	Indirect: 73.9% (Good)
	Washburn, Smith [61]	43.5% (Fair)	82.8% (Excellent)^c^	NR	71.4% (Good)	NR	Indirect: 68.8% (Good)
PWMAQ *Past-7 days*	Pettee Gabriel, McClain [62]	NR	72.4% (Good)^c^	NR	NR	NR	Direct: 69.6% (Good)
	Pettee Gabriel, McClain [38]	NR	71.4% (Good)^c^	NR	NR	NR	Direct: 69.6% (Good) Indirect: 59.1% (Good)
PAR *Past-7 days*	Albanes, Conway [63]	NR	NR	NR	NR	NR	Direct: 30.4% (Fair)
	Blair, Haskell [64]	NR	NR	NR	NR	NR	Direct: 78.3% (Excellent) Indirect: 78.3% (Excellent)
	Conway, Seale [65]	NR	NR	NR	NR	NR	Direct: 69.6% (Good)
	Garfield, Canavan [66]	NR	NR	NR	NR	NR	Direct: 56.5% (Good)
	Gross, Sallis [67]	NR	58.6% (Good)^d^	NR	NR	NR	NR
	Irwin, Ainsworth [68]	NR	NR	NR	NR	NR	Direct: 52.2% (Good) Indirect: 52.2% (Good)
	Johansen, Painter [60]	NR	NR	NR	NR	NR	Direct: 65.2% (Good) Indirect) 56.5% (Good)
	Mahabir, Baer [51]	NR	NR	NR	NR	NR	Direct: 65.2% (Good)
	Rauh, Hovell [52]	NR	65.5% (Good)^c^	NR	NR	NR	Direct: 60.9% (Good) Indirect: 60.9% (Good)
	Sallis, Haskell [69]	36.4% (Fair)	79.3% (Excellent)^c^	NR	NR	NR	Indirect: 73.9% (Good)
	Sarkin, Johnson [70]	NR	NR	NR	NR	NR	Divergent: 34.8% (Fair)
	Taylor, Coffey [71]	NR	NR	NR	NR	NR	Direct: 56.5% (Good) Indirect: 56.5% (Good)
	Washburn, Jacobsen [72]	NR	NR	NR	NR	NR	Direct: 65.2% (Good)
	Williams, Klesges [73]	NR	55.2% (Good)^c^	NR	NR	NR	Direct: 60.9% (Good)
VAPAQ *Past-7 days*	Betz, Myers [74]	NR	58.6% (Good)^c^	62.1% (Good)	NR	NR	NR

^a^Direct comparisons of physical activity measures (e.g. physical activity level between PAQ and other PAQs, diaries or objective measures)

^b^Indirect comparisons of physical activity measures (e.g. physical activity level between PAQ and physical fitness, given the assumption that individuals with greater level of physical activity would have a greater level of physical fitness)

^c^Test-retest reliability

^d^Inter-rater reliability

Table 7 demonstrates the weighted mean of the *r*-values for *test-retest reliability* and *convergent validity* between the types of PAQ (i.e., Usual-week vs. Past-week) and type of comparator measures (i.e., direct vs. indirect measures). Test-retest reliability data was available for 7 of the 20 PAQs. According to the average weighted mean of the *r*-values, the *reliability* of both Usual-week and Past-week PAQs showed strong correlations when assessed across two separate time points, with similar reliability measures for Usual-week (*r* = 0.63) and Past-week (*r* = 0.56) PAQs. According to Cohen’s methods, when direct and indirect measures were combined for *convergent validity* (data was available for 17 of 20 PAQs), Usual-week PAQs exhibited a moderate correlation (*r* = 0.30), whereas Past-week PAQs shows a weak correlation (*r* = 0.28). With respect to measurement type for each recall of PAQ, the *convergent validity* for direct measures had moderate correlations for both Usual-week (*r* = 0.33) and Past-week PAQs (*r* = 0.40) compared to weak correlations for indirect measures (*r* = 0.28 and *r* = 0.19, respectively). When Usual-week and Past-week PAQs were compared separately between direct and indirect measures, similar correlations were observed for the Past-week PAQs (*r* = 0.40) and the Usual-week PAQs (*r* = 0.33) for direct measures with moderate correlations. However, for indirect measures, there was a moderate correlation (*r* = 0.33) for Usual-week PAQs whereas the Past-week PAQs had a weak correlation (*r* = 0.19). Finally, when both Past-week and Usual-week PAQs were combined, direct measures had a moderate correlation (*r* = 0.39) whereas indirect measures had a weak correlation (*r* = 0.21).

**Table 7:** The weighted mean of the *r*-values for reliability testing and convergent validity of Past-week and Usual-week physical activity questionnaires

Instrument	*r*-values	Sample (n)
Reliability testing		
CaMos *Usual-week*	NR	NR
IPEQ-WA *Usual-week*	NR	NR
MAQ *Usual-week*	0.91	46
NHS II *Usual 7-days*	NR	NR
Phone-FITT *Usual-week*	NR	NR
YPAS *Usual 7-days*	0.56	198
AAS *Past 7-days*	NR	NR
AAS (modified) *Past 7-days*	0.65	63
CAQ-PAI *Past 7-days*	0.53	633
Checklist Questionnaire *Past 7-days*	NR	NR
Global Questionnaire *Past 7-days*	NR	NR
IPAQ-LF *Past 7-days*	NR	NR
IPAQ-SF *Past 7-days*	NR	NR
NZPAQ-LF *Past 7-days*	NR	NR
NZPAQ-SF *Past 7-days*	NR	NR
PAAQ *Past 7-days*	NR	NR
PASE *Past 7-days*	0.68	144
PWMAQ *Past 7-days*	NR	NR
PAR *Past 7-days*	0.65	118
VAPAQ *Past 7-days*	0.93	55
Average for Usual-week PAQs	0.63	244
Average for Past-week PAQs	0.56	950
Convergent validity testing		
CaMos		
*Usual-week*		
Direct & Indirect	NR	NR
Direct		
Indirect		
IPEQ-WA		
*Usual-week*		
Direct & Indirect	0.28	553
Direct	0.22	177
Indirect	0.31	376
MAQ		
*Usual-week*		
Direct & Indirect	0.47	118
Direct	0.57	118
Indirect	0.23	66
NHS II		
*Usual 7-days*		
Direct & Indirect	0.27	66
Direct	0.43	66
Indirect	0.22	66
Phone-FITT		
*Usual-week*		
Direct & Indirect	0.36	84
Direct	0.44	48
Indirect	0.25	36
YPAS		
*Usual 7-days*		
Direct & Indirect	0.36	2099
Direct	0.43	824
Indirect	0.34	1182
AAS		
*Past 7-days*		
Direct & Indirect	NR	NR
Direct	NR	NR
Indirect	NR	NR
AAS (modified)		
*Past 7-days*		
Direct & Indirect	0.57	63
Direct	0.57	63
Indirect	NR	NR
CAQ-PAI		
*Past 7-days*		
Direct & Indirect	0.15	3731
Direct	0.46	65
Indirect	0.14	3666
Checklist Questionnaire		
*Past 7-days*		
Direct & Indirect	0.46	2231
Direct	0.46	2231
Indirect	NR	NR
Gobal Questionnaire		
*Past 7-days*		
Direct & Indirect	0.35	2231
Direct	0.35	2231
Indirect	NR	NR
IPAQ-LF		
*Past 7-days*		
Direct & Indirect	0.23	436
Direct	0.23	436
Indirect	NR	NR
IPAQ-SF		
*Past 7-days*		
Direct & Indirect	0.34	25.2
Direct	0.34	25.2
Indirect	NR	NR
NZPAQ-LF		
*Past 7-days*		
Direct & Indirect	NA	NA
Direct	0.25	186
Indirect	NR	NR
NZPAQ-SF		
*Past 7-days*		
Direct & Indirect	NR	NR
Direct	0.25	186
Indirect	NR	NR
PAAQ		
*Past 7-days*		
Direct & Indirect	0.41	318
Direct	0.41	318
Indirect	NR	NR
PASE		
*Past 7-days*		
Direct & Indirect	0.31	355
Direct	0.44	95
Indirect	0.27	260
PWMAQ		
*Past 7-days*		
Direct & Indirect	0.51	64
Direct	0.51	64
Indirect	NR	NR
PAR		
*Past 7-days*		
Direct & Indirect	0.25	3539
Direct	0.39	874
Indirect	0.21	2547
VAPAQ		
*Past 7-days*		
Direct & Indirect	NR	NR
Direct	NR	NR
Indirect	NR	NR
Average for Usual-week PAQs	Direct & Indirect (*r* = 0.30)	Direct & Indirect (*n* = 4730)
	Direct (*r* = 0.33)	Direct (*n* = 2019)
	Indirect (*r* = 0.28)	Indirect (*n* = 2711)
Average for Past-week PAQs	Direct & Indirect (*r* = 0.28)	Direct & Indirect (*n* = 14,147)
	Direct (*r* = 0.40)	Direct (*n* = 6182)
	Indirect (*r* = 0.19)	Indirect (*n* = 7965)
Past-week and Usual-week PAQs	Direct (*r* = 0.39)	Direct (*n* = 8201)
	Indirect (*r* = 0.21)	Indirect (*n* = 10,676)

*Notes*. *CaMos* Canadian Multicentre Osteoporosis Study, *IPEQ-WA* Incidental and Planned Exercise Questionnaire, *MAQ* Modified Activity Questionnaire, *NHS II* Nurses’ Health Study version II, *Phone-FITT* Phone Fitness, *YPAS* Yale Physical Activity Survey, *AAS* Active Australia Survey, *CAQ-PAI* College Alumni Questionnaire – Physical Activity Index, *IPAQ-LF* International Physical Activity Questionnaire Long Form, *IPAQ-SF* International Physical Activity Questionnaire Short Form, *NZPAQ-LF* New Zealand Physical Activity Questionnaire Long Form, *NZPAQ-SF* New Zealand Physical Activity Questionnaire Short Form, *PAAQ* Physical Activity Adult Questionnaire, *PASE* Physical Activity Scale for the Elderly, *PWMAQ* Past Week Modified Activity Questionnaire *PAR* Physical Activity Recall Questionnaire, *VAPAQ* Veterans Physical Activity Questionnaire

Table 8 displays the quality of psychometric properties of both types of PAQs (i.e., Usual-week and Past-week) according to the criteria established by Terwee, Bot [15] and Cordier, Chen [30]. Table 9 provides the overall summary rating of the psychometric properties for each PAQ based on the levels of evidence by Schellingerhout, Verhagen [14]. According to Table 9, very few psychometric properties were reported (40 out of 120 possible ratings: 33.3%), with the quality of psychometric properties primarily reported for *reliability* (13/20: 65%) and *hypothesis testing* (18/20: 90%). Fewer results were identified for *internal consistency* (2/20: 10%), *content validity* (3/20: 15%) and *measurement error* (4/20: 20%), while *structural validity* was not rated for any of the PAQs. Of all the psychometric properties rated for psychometric quality [32], the results were mainly negative (17/40: 42.5%), consisting of “strong negative” (10/40: 25%), “moderate negative” (5/40: 12.5%) and “limited negative” (2/40: 5%). Several psychometric properties were reported with “conflicting” (13/40: 32.5%), whilst fewer psychometric properties were reported for “indeterminate” (5/40: 12.5%). One psychometric property had a “not evaluated” rating, due to poor COSMIN scoring.

**Table 8:** Quality of psychometric properties per study based on the criteria by Terwee, Bot [9] and Cordier, Chen [30]

Assessment	Reference	Measurement properties of questionnaires					
		Reliability				Construct validity	
		Internal Consistency	Reliability	Measurement error	Content validity	Structural validity	Hypothesis testing^ab^
Usual-week Physical Activity Questionnaires							
CaMos *Usual-week*	Nadalin, Bentvelsen [36]	NR	–	NR	NR	NR	NR
IPEQ *Usual-week*	Merom, Delbaere [37]	NR	NR	NR	NR	NR	- (Direct) - (Indirect)
MAQ *Usual-week*	Pettee Gabriel, McClain [38]	NR	–	NR	NR	NR	- (Direct) - (Indirect)
	Kriska, Knowler [39]	NR	+	NR	NR	NR	± (Direct)
	Kriska, Edelstein [40]	NR	NR	NR	NR	NR	- (Direct)
	Schulz, Harper [41]	NR	NR	NR	NR	NR	+ (Direct)
NHS II *Usual-week*	Pettee Gabriel, McClain [38]	NR	–	NR	NR	NR	± (Direct) - (Indirect)
Phone-FITT *Usual-week*	Gill, Jones [32]	NR	+	NR	–	NR	- (Direct) - (Indirect)
YPAS *Usual-week*	Colbert, Matthews [42]	NR	+	?	NR	NR	- (Direct)
	Dipietro, Caspersen [43]	NE	–	?	NR	NR	- (Direct) - (Indirect)
	Gennuso, Matthews [44]	NR	–	NR	NR	NR	? (Direct)
	Harada, Chiu [45]	NR	NR	NR	NR	NR	+ (Direct) - (Indirect)
	Kolbe-Alexander, Lambert [46]	NR	–	?	NR	NR	- (Direct)
	Moore, Ellis [47]	NR	NR	NR	NR	NR	- (Indirect)
Past-week Physical Activity Questionnaires							
Active Australia Survey *Past week*	Brown, Bauman [49]	NR	–	NR	NR	NR	NR
	Brown, Trost [48]	NR	NR	NR	NR	NR	- (Direct)
	Creamer, Bowles [50]	NR	+	NR	NR	NR	- (Direct)
Active Australia Survey (modified) *Past-week*	Fjeldsoe, Winkler [12]	NR	–	?	NR	NR	+ (Direct)
CAQ-PAI *Past-week*	Mahabir, Baer [51]	NR	NR	NR	NR	NR	- (Direct)
	Rauh, Hovell [52]	NR	–	NR	NR	NR	- (Direct) - (Indirect)
	Washburn, Smith [53]	NR	–	NR	NR	NR	- (Indirect) + (Discriminant)
Checklist Questionnaire *Past-week*	Masse, Fulton [54]	NR	NR	NR	NR	NR	± (Direct)
Global Questionnaire *Past-week*	Masse, Fulton [54]	NR	NR	NR	NR	NR	- (Direct)
IPAQ-LF (self-administered) *Past-week*	Ahn, Chmiel [55]	NR	NR	NR	NR	NR	- (Direct)
	Garriguet, Tremblay [56]	NR	NR	NR	NR	NR	- (Direct)
IPAQ-SF(interview) *Past-week*	Ainsworth, Macera [57]	NR	NR	NR	NR	NR	- (Direct)
	Brown, Bauman [49]	NR	–	NR	NR	NR	NR
	Brown, Trost [48]	NR	NR	NR	NR	NR	- (Direct)
NZPAQ-LF *Past-week*	Moy, Scragg [58]	NR	NR	NR	NR	NR	- (Direct)
NZPAQ-SF *Past-week*	Moy, Scragg [58]	NR	NR	NR	NR	NR	- (Direct)
PAAQ *Past-week*	Garriguet, Tremblay [56]	NR	NR	NR	–	NR	- (Direct)
PASE *Past-week*	Colbert, Matthews [42]	NR	–	?	NR	NR	- (Direct)
	Dinger, Oman [59]	NR	+	NR	NR	NR	- (Direct)
	Johansen, Painter [60]	NR	NR	NR	NR	NR	+ (Direct) - (Indirect)
	Moore, Ellis [47]	NR	NR	NR	NR	NR	- (Indirect)
	Washburn, Smith [61]	–	–	NR	+	NR	- (Indirect)
PWMAQ *Past-week*	Pettee Gabriel, McClain [62]	NR	+	NR	NR	NR	+ (Direct)
	Pettee Gabriel, McClain [38]	NR	+	NR	NR	NR	- (Direct) - (Indirect)
PAR *Past-week*	Albanes, Conway [63]	NR	NR	NR	NR	NR	- (Direct)
	Blair, Haskell [64]	NR	NR	NR	NR	NR	- (Direct) - (Indirect)
	Conway, Seale [65]	NR	NR	NR	NR	NR	- (Direct)
	Garfield, Canavan [66]	NR	NR	NR	NR	NR	+ (Direct)
	Gross, Sallis [67]	NR	+	NR	NR	NR	NR
	Irwin, Ainsworth [68]	NR	NR	NR	NR	NR	- (Indirect)
	Johansen, Painter [60]	NR	NR	NR	NR	NR	+ (Direct) - (Indirect)
	Mahabir, Baer [51]	NR	NR	NR	NR	NR	- (Direct)
	Rauh, Hovell [52]	NR	–	NR	NR	NR	- (Direct) - (Indirect)
	Sallis, Haskell [69]	?	–	NR	NR	NR	- (Indirect)
	Sarkin, Johnson [70]	NR	NR	NR	NR	NR	? (Indirect)
	Taylor, Coffey [71]	NR	NR	NR	NR	NR	+ (Direct)
	Washburn, Jacobsen [72]	NR	NR	NR	NR	NR	- (Direct) - (Indirect)
	Williams, Klesges [73]	NR	+	NR	NR	NR	± (Direct)
VAPAQ *Past-week*	Betz, Myers [74]	NR	+	?	NR	NR	NR

*Notes*. ^a^Direct comparisons of physical activity measures (e.g., physical activity level between PAQ and other PAQs, diaries or objective measures)

^b^Indirect comparisons of physical activity measures (e.g., physical activity level between PAQ and physical fitness, given the assumption that individuals with greater level of physical activity would have a greater level of physical fitness)

**Table 9:** Overall quality score of psychometric properties for each interview-administered Usual-week and Past-week physical activity questionnaire using the levels of evidence by Schellingerhout et al., [14]

Assessment	Internal Consistency	Reliability	Measurement error	Content validity	Structural validity	Hypothesis testing
CaMos *Usual-week*	NR	Strong Negative	NR	NR	NR	NR
IPEQ *Usual-week*	NR	NR	NR	NR	NR	Moderate Negative
MAQ *Usual-week*	NR	Conflicting	NR	NE	NR	Conflicting
NHS II *Usual-week*	NR	Moderate Negative	NR	NR	NR	Conflicting
Phone-FITT *Usual-week*	NR	Moderate Positive	NR	Limited Negative	NR	Moderate Negative
YPAS *Usual-week*	Not Evaluated	Conflicting	Indeterminate	NR	NR	Conflicting
AAS *Past-7 days*	NR	Conflicting	NR	NR	NR	Strong Negative
AAS (modified) *Past-7 days*	NR	Strong Negative	Indeterminate	NR	NR	Strong Positive
CAQ-PAI *Past-7 days*	NR	Strong Negative	NR	NR	NR	Conflicting
Checklist Questionnaire *Past-7 days*	NR	NR	NR	NR	NR	Conflicting
Global Questionnaire *Past-7 days*	NR	NR	NR	NR	NR	Moderate Negative
IPAQ-LF (self-administered) *Past-7 days*	NR	NR	NR	NR	NR	Strong Negative
IPAQ-SF (interview) *Past-7 days*	NR	Strong Negative	NR	NR	NR	Strong Negative
NZPAQ-LF *Past-7 days*	NR	NR	NR	NR	NR	Strong Negative
NZPAQ-SF *Past-7 days*	NR	NR	NR	NR	NR	Strong Negative
PAAQ *Past-7 days*	NR	NR	NR	Moderate Negative	NR	Strong Negative
PASE *Past-7 days*	Limited Negative	Conflicting	Indeterminate	Moderate Positive	NR	Conflicting
PWMAQ *Past-7 days*	NR	Strong Positive	NR	NR	NR	Conflicting
PAR *Past-7 days*	Indeterminate	Conflicting	NR	NR	NR	Conflicting
VAPAQ *Past-7 days*	NR	Moderate Positive	Indeterminate	NR	NR	NR

*Notes*. *CaMos* Canadian Multicentre Osteoporosis Study, *IPEQ-WA* Incidental and Planned Exercise Questionnaire, *MAQ* Modified Activity Questionnaire, *NHS II* Nurses’ Health Study version II, *Phone-FITT* Phone Fitness, *YPAS* Yale Physical Activity Survey, *AAS* Active Australia Survey, *CAQ-PAI* – College Alumni Questionnaire – Physical Activity Index; *IPAQ-LF* International Physical Activity Questionnaire Long Form, *IPAQ-SF* International Physical Activity Questionnaire Short Form, *NZPAQ-LF* New Zealand Physical Activity Questionnaire Long Form, *NZPAQ-SF* New Zealand Physical Activity Questionnaire Short Form, *PAAQ* Physical Activity Adult Questionnaire, *PASE* Physical Activity Scale for the Elderly, *PWMAQ* Past Week Modified Activity Questionnaire, *PAR* Physical Activity Recall Questionnaire, *VAPAQ* Veterans Physical Activity Questionnaire

The relative number of negative (“strong negative” [6/18: 33.3%] and “moderate negative” [3/18: 16.7%]) and conflicting (8/18: 44.4%) ratings were reported the most for *hypothesis testing*; only one “strong positive” rating was identified. For *reliability*, a greater relative number of positive ratings (“strong positive” [1/13: 7.7%] and “moderate positive” [2/13: 15.4%]) were found. However, *reliability* also exhibited several negative (“strong negative” [4/13: 30.8%] and “moderate negative” [1/13: 7.7%]) and conflicting (5/13: 38.5%) ratings. The relative number of “indeterminate” ratings was greatest for *measurement error* (4/4: 100%), whilst *internal consistency* showed only one “indeterminate”, “limited negative” and “not evaluated” ratings (1/3: 33.3%, respectively). There was one “moderate positive” rating (1/3: 33.3%) for *content validity*, although the rest of the ratings consisted of one “moderate negative” and “limited negative” ratings (1/3: 33.3%, respectively).

When comparing the PAQs, *reliability* demonstrated positive ratings for Past-Week Modifiable Activity Questionnaire (PWMAQ) (“strong positive”), Phone-FITT (“moderate positive”) and the Veterans Physical Activity Questionnaire (VAPAQ) (“moderate positive”). However, the results for PWMAQ also demonstrated a “conflicting” rating in *hypothesis testing*, Phone-FITT received a “limited negative” and “moderate negative” in *content validity* and *hypothesis testing*, respectively, and VAPAQ received an “indeterminate” rating for *measurement error*. The AAS (modified) demonstrated a “strong positive” rating for *hypothesis testing*, although this PAQ also received a “strong negative” and “indeterminate” rating for *reliability* and *measurement error*, respectively. While PWMAQ, Phone-FITT, VAPAQ and AAS (modified) received mixed results, these measures have a substantial number of psychometric properties that were not reported.

When compared between the types of PAQs, a similar percentage of negative ratings (limited, moderate or strong) were shown for Past-week (12/28: 42.9%) and Usual-week (5/12: 41.7%) PAQs. Conversely, the relative number of positive ratings (limited, moderate or strong) for the Past-week PAQs (4/28: 14.3%) was greater than Usual-week PAQs (1/12: 8.3%), although the absolute number of “positive” ratings were small. The number of NR ratings [33] were noticeable across all PAQs.

## Discussion

This systematic review examined the methodological quality of studies that investigated the psychometric properties of interview-administered, Usual-week and Past-week PAQs, in an adult population. There were 20 PAQs with 42 corresponding articles that reported on the psychometric properties of PAQs, of which 6 were Usual-week and 14 were Past-week PAQs. Amongst the psychometric properties, *hypothesis testing* was reported most frequently, followed by *reliability*, whereas *measurement error*, *content validity* and *internal consistency* were the least reported. Furthermore, *structural validity* was not reported in any of the included studies. The methodological quality of the studies exhibited good to excellent ratings across most of the psychometric properties. As per the average weighted mean of the *r*-values, both Usual-week PAQs and Past-week PAQs showed moderate correlations for *reliability* and *convergent validity* for direct measures, whereas *convergent validity* for indirect measures exhibited weak correlations irrespective of the type of PAQ. When comparing the weighted mean of the *r*-values between PAQ types, *convergent validity* for direct measures indicated moderate correlations for both Past-week and Usual-week PAQs, although *convergent validity* for indirect measures demonstrated moderate correlations for Usual-week PAQs, while weak correlations were observed for Past-week PAQs. According to the level of evidence (i.e., overall quality), most of the psychometric properties exhibited “moderate negative” to “strong negative” ratings irrespective of PAQ types, highlighting concerns for utilising current interview-administered PAQs.

### Quality of studies based on the COSMIN taxonomy

Of the psychometric property reliability, most psychometric studies reported on *test-retest reliability* with good to excellent COSMIN ratings, whereas *measurement error* was only reported for four measures (YPAS, AAS [modified], PASE and VAPAQ), also with good to excellent ratings. *Measurement error* is an essential property of the reliability dimension, as it quantifies the magnitude of systematic and random error of PA levels that is not caused by true changes in the construct being measured; thus allowing practitioners to establish meaningful differences in PA measures [34]. Subsequently, more research is warranted to determine the *measurement error* of PAQs when administered in an interview setting. Compared to *test-retest reliability* and *measurement error*, *internal consistency* was reported for only three measures (YPAS, PASE and PAR) with poor to fair ratings. This discrepancy was due to included studies consisting of a small sample size and/or examining correlations between different items of the same PAQ without conducting Cronbach alpha statistic and factor analyses. According to Terwee et al. [13], both statistical approaches determine whether all items measure the same construct and checks the uni-dimensionality of the scale. Thus, future studies should consider these limitations when examining the *internal consistency* of interview-administered PAQs.

With the exception of two PAQs (CaMos and VAPAQ), *hypothesis testing* was reported for all PAQs with the majority of included studies reporting good to excellent ratings for methodological quality. However, studies only investigated three PAQs (Phone-FITT, PAAQ and PASE) for *content validity* with fair to good COSMIN ratings. The methodological limitations identified from these studies included lack of description on whether piloting was conducted by investigators, expert practitioners and/or the target population. Thus, future studies should consider these issues when examining the *content validity* of PAQs. Most alarming is that none of the PAQs investigated *structural validity*. This means that the underlying constructs of all the PAQs are currently unknown, as appropriate statistical analyses to ascertain the factor structure or dimensionality (e.g., dimensionality and principle component analysis using Rasch analysis and exploratory and/or confirmatory factor analysis) of the measures and associated subscales have not been conducted. Therefore, when assessing *structural validity* of PAQs, future studies should identify whether their approach is in accordance with a formative (i.e., integrative items forming a construct) or reflective (i.e., items are reflective of the same underlying constructs) model.

### Quality of psychometric properties

In the current review, *test-retest reliability* for the Usual-week PAQs was comparable to the Past-week PAQs based on the average weighted mean of the *r*-values. These results are in contrast to a previous study by Delbaere, Hauer [35], who compared the reliability of self-administered incidental and planned exercise questionnaire (IPEQ) between Usual-week (i.e., past three months) and Past-week versions. According to their results, the Usual-week IPEQ (*ICC* = 0.84) version showed greater *test-retest reliability* compared to the Past-week IPEQ version (*ICC* = 0.77). Authors speculated that PAQs with usual 7-day recall periods during the past several months exhibit better stability in PA measures across time points, compared with PAQs with past-week recalls given that PA levels may fluctuate from week-to-week [10], or season-to-season [11]. The discrepancies in findings between the current review, and the work by Delbaere, Hauer [35], may be due to distinct acceptable cut-off levels being employed for *test-retest reliability*. For example, Delbaere, Hauer [35] established acceptable ICC values at ≥0.6, whereas the current review utilised an acceptable ICC criteria of ≥0.7 according to the criteria set out by [15]. Therefore, where ICC values (≥0.6 to < 0.7) were classified as “acceptable” for [35], would have been considered below the acceptable cut-off level in the current review with a “negative” rating. In addition, the *test-retest reliability* in the current review was compared between PAQs with different recall methods based on average weighted mean of the *r*-values across multiple studies, whereas [35] compared different recall versions of IPEQ within the same study and population. Subsequently, the variation in study design and the type of PAQs may have diluted potential differences in the weighted mean of the *r*-values between Past-week and Usual-week PAQs in the current review. This is further supported by a previous systematic review by [16], who also reported comparable *test-retest reliability* of average weighted mean of the *r*-values for self-administered Usual-week and Past-week PAQs.

When comparing *convergent validity* (i.e., *hypothesis testing*) between PAQ recall types, the average weighted mean of the *r*-values of Past-week PAQs were comparable with Usual-week PAQs for direct measures. However, the average weighted mean of the *r*-values was greater for Usual-week PAQs compared with Past-week PAQs within our review. These findings differ to a previous systematic review reported by [16] in self-reported PAQs where *convergent validity* for direct measures were greater for Past-week PAQs than Usual-week PAQs. In addition, the average weighted mean of the *r*-values for direct measures of *convergent validity* for both PAQ recall periods showed a moderate correlation (*r* = 0.35), whereas Doma, Speyer [16] reported weak correlations for the same measures (*r* = 0.27) based on self-reported PAQs in their previous review. This trend has also been reported by previous studies that compared *convergent validity* between interview-administered and self-administered PAQs [36, 37]. For example, Chu, Ng [36] reported stronger associations between Global Physical Activity Questionnaire (GPAQ) for the interview-administered method (*r* = 0.44–0.52) compared with the self-administered method (*r* = 0.28–0.38) when compared against accelerometers. Collectively, PAQs administered via interview may allow reporting of PA levels with greater accuracy than by self-administration, possibly due to minimisation of respondent bias [36].

While weighted-mean of the *r*-values for direct measures of *convergent validity* were comparable between Past-week and Usual-week PAQs, indirect measures of *convergent validity* were stronger for Usual-week PAQs (i.e., moderate correlations) than Past-week PAQs (i.e., weak correlations). This suggests that Usual-week PAQs better reflect physical fitness (e.g., VO_2max_, 6-min walk test) and its associated physiological conditions (e.g., BMI, body fat percentage) than Past-week PAQs when administered via interviews. These results are expected, given that physical fitness measures are stable across several weeks despite exercise termination [38], as opposed to the inherent week-to-week fluctuations observed with PA level [39]. Subsequently, when estimating physical fitness levels based on PA level ascertained from PAQs, we encourage the use of Usual-week PAQs rather than Past-week PAQs, particularly when administered via interviews. However, it should be noted that the current review included studies consisting of older adults with a number of pathological conditions (e.g., cardiovascular disease, musculoskeletal disease and neurological disease), where chronic exercise adaptations and deconditioning may differ in response to apparently healthy, younger individuals [32, 40, 41]. Separating these populations was difficult in the current review as the majority of studies incorporated apparently healthy participants with those who had several pathological conditions in the one study. Thus, future research should systematically compare psychometric properties of PAQs between individuals with pathological conditions and their apparently healthy counterparts.

For the overall Level of Evidence, irrespective of recall methods, there was a substantial number of missing psychometric data (i.e., not reported [NR]), indicating that the psychometric properties pertinent to determining the quality of current PAQs are not being examined effectively. Of the few psychometric properties reported, there were only four that reported “moderate positive” to “strong positive” ratings, with the rest as “strong to limited negative”, “indeterminate” and “conflicting” ratings, which demonstrate the weaknesses of current PAQs. No studies examined *structural validity* of PAQs, and only three PAQs examined *internal consistency* with ratings of “not evaluated”, “indeterminate” and “limited negative”. These weak results and lack of reporting is particularly concerning given that both *structural validity* and *internal consistency* are based on a reflective model, whereby all items are manifestations of the same underlying construct [27]. In addition, only a very limited number of PAQs reported on *content validity* (3/20: 15%), with one “positive” rating and two “negative” ratings. These findings further raise the limitations of current interview-administered PAQs, as *content validity* measures the degree to which the content of a PAQ is an adequate reflection of the construct being measured [27].

When comparing the overall psychometric qualities of PAQs based on Level of Evidence between recall methods, there were minute differences between Usual-week and Past-week PAQs, which are in line with findings by [16]. Additionally, the “moderate negative” to “strong negative” ratings shown for the majority of psychometric properties in the current review are similar to those reported by other systematic reviews [16, 42, 43]. These psychometric properties were rated poorly as the correlations were predominantly below the acceptable levels for *test-retest reliability* and *convergent validity*. However, authors from several studies included in the current review reported that the PAQs demonstrated acceptable *test-retest reliability* and *convergent validity*, which conflicts with findings from this current review. The discrepancy in these interpretations is because authors in the included studies considered *test-retest reliability* and *convergent validity* as acceptable based on level of significance (*p* ≤ 0.05), rather than the strength of the relationship (i.e., magnitude of the *r*-values). Accordingly, the strength of the relationship should be accounted for by future studies, as larger sample sizes are likely to generate associations at a statistically significant level, irrespective of the strength of the relationship. While the methodological quality of *measurement error* was rated as “good” to “excellent”, the four PAQs corresponding to these ratings (i.e., YPAS, AAS [modified], PASE and VAPAQ) were classified as “indeterminate” for psychometric quality. This is because the included studies did not report minimal important change (MIC) with respect to smallest detectable change (SDC), or whether MIC ranged beyond the limits of agreement (LOA). According to Terwee, Roorda [44], SDC and MIC are essential parameters for reliability to allow better interpretation of change scores. Subsequently, more studies need to incorporate *measurement error* when examining reliability of PAQs and consider calculations of MIC and SDC and/or LOA for this psychometric property.

### Limitation

The primary purpose of the current review was to examine the psychometric properties of interview-administered, Past-week and Usual-week PAQs in an adult population. Thus, investigating the psychometric properties of PAQs with recall time-frames beyond, or within, the 7-day period was beyond the scope of the study. In addition, the current review selectively included studies that examined the psychometric properties of PAQs that were published in an English-speaking country because cultural diversity appears to impact on the psychometric properties of PAQs (e.g., errors of translation between languages, interpretation difficulties). Furthermore, the current review specifically selected studies that were conducted in an adult population, given that PAQs for children and adolescents are developed according to their literacy level. Thus, comparing the psychometric qualities of PAQs between studies that were conducted in English-speaking and non-English speaking countries and between age groups (i.e., children, adolescents and adults) may expand our knowledge on the usability of PAQs across different population groups. Whilst we made every effort to exclude studies that included participants with diagnosed cognitive impairment, the majority of the included studies did not screen for cognitive impairment. Therefore, future studies should consider conducting cognitive assessments to ensure that cognitive conditions are not influencing the psychometric properties of PAQs, particularly in older adults. With respect to abstract screening, there were discrepancies between those who conducted the literature search and those who screened the abstracts, which may have introduced selective bias. However, the reviewers were rigorously trained prior to abstract screening to ensure transparency of the inclusion criteria, and any disagreement between reviewers were resolved by the primary author (KD). Finally, examining the *responsiveness* and *cross-cultural validity* of PAQs was beyond the scope of this review. Therefore, comparing the psychometric quality of these properties between different PAQ types may allow better understanding of the sensitivity to changes in PA level.

## Conclusion

The current review demonstrated that the psychometric quality of the majority of reported psychometric properties exhibited “negative” ratings. In addition, minimal differences were identified in the psychometric quality between Usual-week and Past-week PAQs. These findings suggested that the psychometric qualities of commonly used interview-administered PAQs are weak irrespective of recall methods. Therefore, caution should be used when measuring PA level using the PAQs included in this review. According to the weighted mean of the *r*-values, *test-retest reliability* was stronger for Usual-week PAQs compared with Past-week PAQs, although the reverse was identified for *convergent validity* for direct measures of PA level. These results indicate that Usual-week PAQs may be more suitable when identifying PA levels, and its corresponding association with physical fitness, of a large population for epidemiological studies. Conversely, Past-week PAQs may allow better detection of changes in PA level following an intervention. Finally, the interview-administered PAQs exhibited stronger *convergent validity* than previously reported for self-administered PAQs [16]. Therefore, whilst interview-administered PAQs may be time-consuming and cumbersome, researchers may opt to utilise this method over self-administered PAQs to obtain greater accuracy in physical activity level. However, irrespective of the strength of correlations, it is important to note that the quality of the measurement properties were either not examined or were quite poor. Subsequently, future studies should investigate the psychometric properties using more robust methodologies based on the COSMIN to better understand the usability of current PAQs, or to develop new PAQs by addressing issues identified in this review.

## Abbreviations

AAS: Active Australia Survey
CaMos: Canadian Multicentre Osteoporosis Study
CAQ-PAI: College Alumni Questionnaire – Physical Activity Index
COSMIN: Consensus-based Standards for the Selection of Health Measurement Instrument
ICC: Intra-class correlation coefficient
IPAQLF: International Physical Activity Questionnaire Long Form
IPAQ-SF: International Physical Activity Questionnaire Short Form
IPEQ-WA: Incidental and planned exercise questionnaire
LOA: Limits of agreement
MAQ: Modified activity questionnaire
MET: Metabolic equivalent of task
MIC: Minimal important change
NE: Not evaluated
NHS II: Nurses’ Health Study version II
NR: Not reported
NZPAQ-LF: New Zealand Physical Activity Questionnaire Long Form
NZPAQ-SF: New Zealand Physical Activity Questionnaire Short Form
PA: Physical activity
PAAQ: Physical activity adult questionnaire
PAQ: Physical activity questionnaire
PAR: Physical activity recall questionnaire
PASE: Physical activity scale for the elderly
Phone-FITT: Phone fitness
PRISMA: Preferred reporting items for systematic reviews and meta-analyses
PWMAQ: Past week modified activity questionnaire
SCD: Smallest detectable change
VAPAQ: Veterans Physical Activity Questionnaire
WHO: World health organisation
YPAS: Yale Physical Activity Survey
